# Synthesis and Characterization of Eco-Friendly Bio-Composite from Fenugreek as a Natural Resource

**DOI:** 10.3390/polym14235141

**Published:** 2022-11-25

**Authors:** Nayem Hossain, Mohammad Asaduzzaman Chowdhury, Tauhidul Islam Noman, Md. Masud Rana, Md. Hasan Ali, Raja Saad Alruwais, Md. Shafiul Alam, Khalid A. Alamry, Mahmood D. Aljabri, Mohammed M. Rahman

**Affiliations:** 1 Department of Mechanical Engineering, IUBAT-International University of Business Agriculture and Technology, Dhaka 1230, Bangladesh; 2 Department of Mechanical Engineering, Dhaka University of Engineering and Technology (DUET), Gazipur 1700, Bangladesh; 3 Chemistry Department, Faculty of Science and Humanities, Shaqra University, Dawadmi 11912, Saudi Arabia; 4 Chemistry Department, King Abdulaziz University, P.O. Box 80203, Jeddah 21589, Saudi Arabia; 5 Department of Chemistry, University College in Al-Jamoum, Umm Al-Qura University, Makkah 21955, Saudi Arabia; 6 Center of Excellent for Advanced Materials Research (CEAMR) & Department of Chemistry, King Abdulaziz University, P.O. Box 80203, Jeddah 21589, Saudi Arabia

**Keywords:** starch bio-composite, environmental concern, natural resource, biodegradability, antimicrobial activity

## Abstract

The present study show the usability of starch (tamarind) based-bio-composite film reinforced by fenugreek by various percentages to replace the traditional petrochemical plastics. The prepared bio-composite films were systematically characterized using the universal testing machine (UTM), soil degradation, scanning electron microscope (SEM), X-ray diffraction (XRD), thermogravimetric analyzer (TGA), and antibacterial tests. The experiments showed that a lower percentage of fenugreek improves biodegradation and mechanical strength. More than 60% of biodegradation occurred in only 30 days. Almost 3 N/mm^2^ tensile strength and 6.5% tensile strain were obtained. The presence of micropores confirmed by SEM images may accelerate the biodegradation process. Antibacterial activity was observed with two samples of synthesized bio-composite, due to photoactive compounds confirmed by FTIR spectra. The glass transition temperature was shown to be higher than the room temperature, with the help of thermal analysis. The prepared bio-composite containing 5% and 10% fenugreek showed antibacterial activities.

## 1. Introduction

Our everyday human life has been transformed by plastic playing an important part in many aspects of life. Global plastic production from petrochemical sources is increasing rapidly, owing to its extraordinary mechanical, versatility, and barrier properties [1]. As a raw material, around 4% of the total extracted fossil fuels are consumed for the production of these bio-composites. Increasing future demand suggests that by the year 2050, 20% of total fossil fuel extracted internationally may be consumed to produce plastic [2]. The production of these plastics is creating a big challenge, as very few of them are recycled or reused [3,4]. These plastics can remain in the environment for a long period of time, even 1000 years. Moreover, the harmful effect of this waste on the environment is very high. A significant amount of carbon dioxide and other toxic gases are released from this waste, which is harmful to human health and nature [5]. Because of these effects on human health and the environment, finding an alternative has become inevitable.

Bio-composite can be a suitable alternative to petrochemical plastic. Significant development is being made in bio-composite, to make it usable. The production of these plastics has also significantly increased, although the production of bio-composites is only 0.59% of total plastic production [6,7]. The data suggest that more research is necessary to replace harmful plastics completely in any kind of application. One big drawback of bio-composite is its high price compared to conventional plastic, which makes it uncompetitive in the market and limits its use. One alternative to reduce the cost can be the use of waste or by-products of the agri-food industry, which are produced every day in huge quantities [8]. This low-cost waste and by-products are rich in protein, making them competitive candidates to be used as raw material to synthesize bio-composite [9].

The materials of bio-composite that need to biodegrade quickly should have antibacterial properties to kill harmful viruses and bacteria produced from food, but should not have harmful effects on human health and the environment, in order to be used as biodegradable food packaging. Many researchers have synthesized composite bio-composite to improve physio-chemical properties, but such a type of bio-composite contains harmful additives, such as sulfuric acid or titanium dioxide [10,11,12,13,14]. Thus, it is necessary to focus on the research of synthesizing biodegradable bio-secured plastic that is not harmful to human beings and the environment, is not expensive, has antibacterial properties, is available in nature, and represents properties allowing it to be used as a true alternative to petrochemical plastic.

Starch is an available, biodegradable, and low-cost material used as a renewable polymer in wide number of applications. The production of biodegradable films using starch shows promising results, although shortcomings are still there in mechanical properties, dimensional stability, hydrophilicity, and light permissibility. A nonstructured and plasticized version of starch is known as thermoplastic starch, prepared by adding plasticizers to the mixture of starch. Plasticizers can penetrate starch molecules and form hydrogen bonds, which are necessary to increase the durability of the bio-composite [14]. Thermoplastic starch materials are cost-effective, biodegradable, abundant in nature, and renewable. However, humidity causes recrystallization problems in these materials, which drastically decreases their mechanical properties [15]. The other shortcomings of these materials are their hydrophilic character and lower thermal stability [16,17].

Throughout human history, it has been known that Fenugreek is consumed as a food and used as medicine. Its seeds are used in spices, to increase the taste of food. Numerous medicinal properties such as hypocholesterolemic, gastric stimulant, antidiabetic agent, hepatoprotective effect, lactation aid, anticancer, galactagogue, and antibacterial, are available in the seeds of fenugreek. Some of these effects are attributed to the intrinsic dietary fiber constituent. The texture of food is changed by the dietary fiber, which is almost 25% of the seeds. It is also used as a food stabilizer, emulsifying agent, and adhesive, because of its high fiber, gum, and protein content. The protein is more soluble in an alkaline solution. It helps us with digestion, and it can modify food [18,19]. Fenugreek contains up to 60% starch. Different industrial products such as polysaccharides, kernel powder, gum, starch, and oil are produced (Table 1) from fenugreek [20,21,22].

Tamarind is a commercially valuable plant that grows in different parts of Asia, Africa, and America. It is an evergreen plant belonging to the Fabaceae family and Caesalpinioideae subfamily. Different parts of the tree, including leaves, seed, shell, and fiber are used in pharmaceutical, food, electrochemical, biofuel, composite, water, and textile industries [23]. Around 55% pulp, 34% seed, and 11% shell and fiber are available in a typical tamarind pod. Tartaric acid, reducing sugar, and minerals including calcium, phosphorus, and potassium are available in the pulp of tamarind seeds [24]. The pulp has antimicrobial properties and can be used as a preservative. Tamarind seeds contain Zn, Fe, Mg, P, Na, K and Ca as minerals [25]. Tamarind seeds contain polysaccharides that are naturally biodegradable and biocompatible. A typical tamarind fruit fiber contains cellulose, hemicelluloses, lignin, wax, and moisture. The tamarind shell which covers the pulp contains carbohydrates, free tartaric acid, and protein [26]. The leaves of tamarind are composed of lipids, vitamins, fatty acids, and flavonoids [27]. Table 2 shows the percentages of chemical compounds present in the tamarind seed [28].

In the current situation of increased pollution worldwide because of synthetic petrochemical plastic, biodegradable composite can be a good source that will help to minimize environmental pollution. Both tamarind and fenugreek are abundant in the local area, and can be grown in vast quantities because of the good quality of the soil. Therefore, it can be said that both tamarind and fenugreek can be used as raw materials to manufacture bio-composite as an alternative source.

The current research work shows the synthesis and characterization of biodegradability properties of bio-composites synthesized from naturally available and cheap sources, which can be used as an alternative to synthetic plastic. The purpose of this work is to show the usability of naturally cheap sources of a biodegradable composite material that can kill bacteria, so that the material can be used for food-packaging applications. The bio-composite in this research work was synthesized from naturally available fenugreek seeds. The synthesized bio-composites were characterized by biodegradable, mechanical, FTIR, SEM, XRD, thermal and antimicrobial tests. 

## 2. Materials and Methodology

### 2.1. Materials

The bio-composites were synthesized by tamarind, fenugreek, distilled water, vinegar, and glycerin. After collecting the tamarind seeds from the local market of the Gazipur district, Bangladesh, they were washed properly with deionized water, boiled for 30 min, and blended to make the extract of starch. Fenugreek was also collected from the local market of the Gazipur district of Bangladesh. The collected fenugreek was also washed properly with deionized water, boiled for 30 min, and blended, and thus starch was obtained. The environmental lab of IUBAT within the department of civil engineering supplied the necessary distilled water for the experiments. Glycerin and white vinegar were also collected from the local market of the Gazipur District of Bangladesh.

### 2.2. Production of Bio-Composite

**Figure 1:**
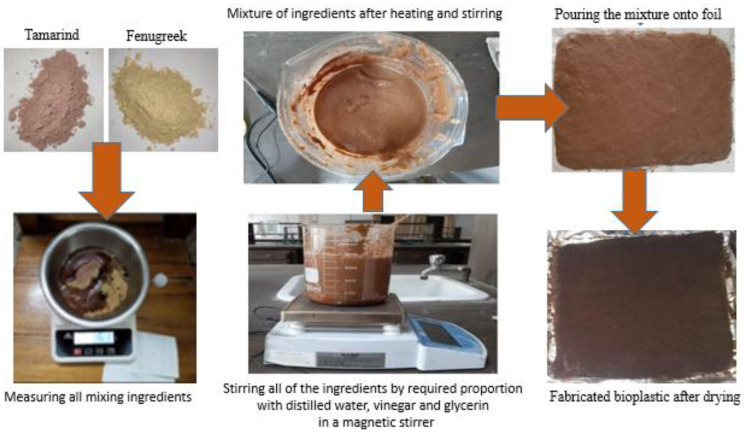
Bio-composite preparation from the natural sources.

Table 3 shows the synthesized bio-composites at different percentages of fenugreek. Initially, with the help of a precise electronic scale, all the ingredients were carefully and precisely weighted. After measuring, the mixing of the ingredients was performed using a magnetic stirrer shown in Figure 1, followed by blending. Clumping was avoided by stirring for seven minutes. Heat was applied to the process at 100 °C temperature. A thick and translucent mixture was obtained after some time. Aluminum foil was used for pouring the mixture, and bubbles were removed if found. The desired bio-composites were obtained after six hours of natural cooling. The thickness of the obtained bio-composite was 1 mm, and it was opaque and chocolate-colored. The obtained bio-composite samples were then taken for characterization. All the samples were made in a dry environment.

### 2.3. Characterization

#### 2.3.1. Biodegradation Test

The synthesized bio-composites were subjected to different characterization processes. For the biodegradability test, each sample was cut to a size of 50 mm × 20 mm × 1 mm. The average weight of each sample was 10 gm. The samples were buried at 2 cm depth. The pH value of the soil was 6. Weight loss was measured by burying the bio-composite samples in soil for 7, 15, and 30 days, in aerobic conditions. Before burying the bio-composite samples under the soil, the weight of each sample was measured carefully, using a precise electronic balance. After the test, each sample was removed from the soil, cleaned with water, dried, and the weight was taken again (Table 3). The biodegradability was measured using the following formula:

$$\mathrm{Biodegradability}\;\left(\%\right)=\frac{W1-W2}{W1}\times 100$$

Here, *W*1 = the weight of the bio-composite sample before the biodegradable test.

*W*2 = the weight of the bio-composite sample after the biodegradable test [29,30,31,32,33,34].

#### 2.3.2. Mechanical Test

After production, the bio-composite samples were taken for mechanical testing. A universal testing machine controlled by a computer called CMT-10 was employed to evaluate the tensile properties of the bio-composite samples. All the tests were conducted maintaining the ASTM D638-77 standard method. The samples were cut with a dimension of 100 mm × 30 mm, in a dry environment. Then the samples were hung on a ring, using a thread at the bottom part of the samples with an attached hook, to place the loads. Maintaining a 2 mm/min strain rate, the force–distance data was measured at room temperature, and the loads were applied until the samples failed. The total length of the failure samples was measured carefully, and recorded. The total applied loads were recorded as well. Elongation and tensile strength were measured with the help of a stress–strain curve. For the same condition, 5 experiments were done for each sample, and the average value was considered.

#### 2.3.3. Scanning Electron Microscopy Test

The surface microstructure of the synthesized bio-composites was analyzed by a Hitachi brand scanning electron microscope, model number S-4800. For analyzing the surface of these bio-composite samples, the bio-composites were submerged in liquid nitrogen and cut into 0.5 cm^2^-sized samples. Then, cryo-fracturing was performed. The cryo-fractured samples were fixed onto the support using adhesive tape and mounted on aluminum stubs. Coating of the bio-composite samples was performed with gold-palladium, to observe the microstructure.

#### 2.3.4. X-ray Diffraction Test

The X-ray diffraction analysis of the synthesized bio-composites was performed using a Rigaku, Tokyo, Japanese-made X-ray diffractometer, where scattering speed was 0.02(θ) s^−1^ ranging 5 to 60° (2θ) angles maintaining 40 kV voltage and 35 mA current, to determine the crystallographic structure.

#### 2.3.5. Thermal Analysis

An SDT 650 TA-Instrument was employed to analyze the TGA of the synthesized bio-composites at a heating rate of 5 °C and weighing 10–25 mg, from room temperature to 500 °C temperature. TGA was performed in a nitrogen environment, with the lid kept hermetically sealed.

#### 2.3.6. Antimicrobial Test

The evaluation of the antimicrobial properties of the developed bio-composite samples was performed using the ASTM-E2149-01 standard. Here, the evaluation of microbial growth and the resistivity of the non-leaching antimicrobial-treated specimens was performed under dynamic conditions. The microbiology department of Dhaka University supplied the necessary microbial for the experiments. The Kirby–Bauer disk-diffusion method was used to examine the antimicrobial performance of the synthesized bio-composites. *Staphylococcus aureus* was the bacteria cell subjected to the microbial test. The bacterial cell and sample concentrations were 1000 CFU/mL and 200 mg/mL, respectively, and the disc size was 6 mm × 6 mm.

## 3. Results and Discussion

### 3.1. Biodegradation

**Figure 2:**
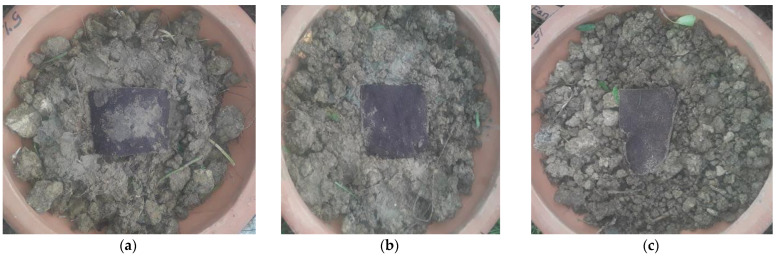
Prepared samples for soil biodegradation test (**a**) 5% fenugreek, (**b**) 10% fenugreek, and (**c**) 15% fenugreek.

**Figure 3:**
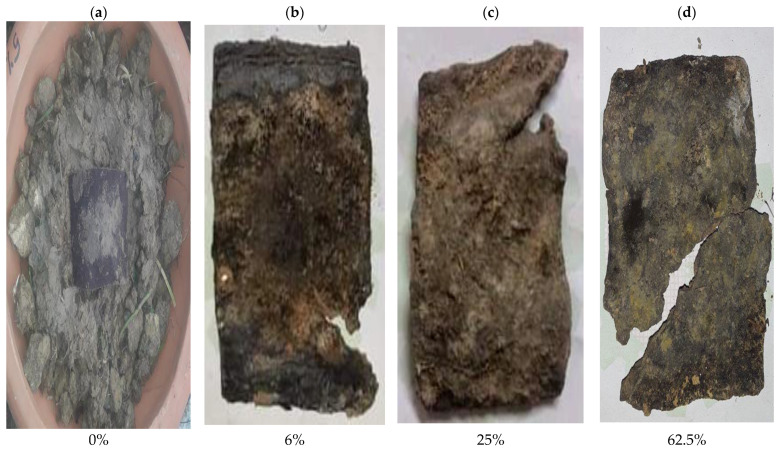
5% fenugreek containing bio-composite degradation sample on (**a**) Day 1, (**b**) Day 7, (**c**) Day 15, and (**d**) Day 30.

**Figure 4:**
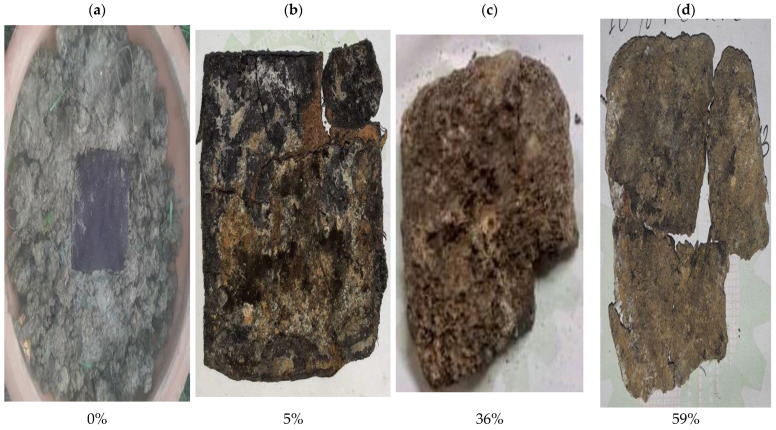
10% fenugreek containing bio-composite degradation sample on (**a**) Day 1, (**b**) Day 7, (**c**) Day 15, and (**d**) Day 30.

**Figure 5:**
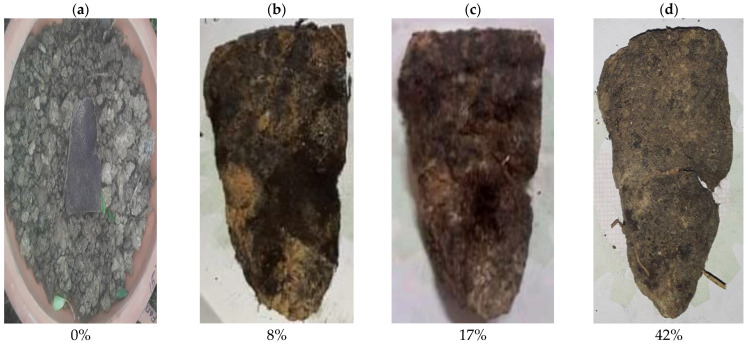
15% fenugreek containing bio-composite degradation sample on (**a**) Day 1, (**b**) Day 7, (**c**) Day 15, and (**d**) Day 30.

**Figure 6:**
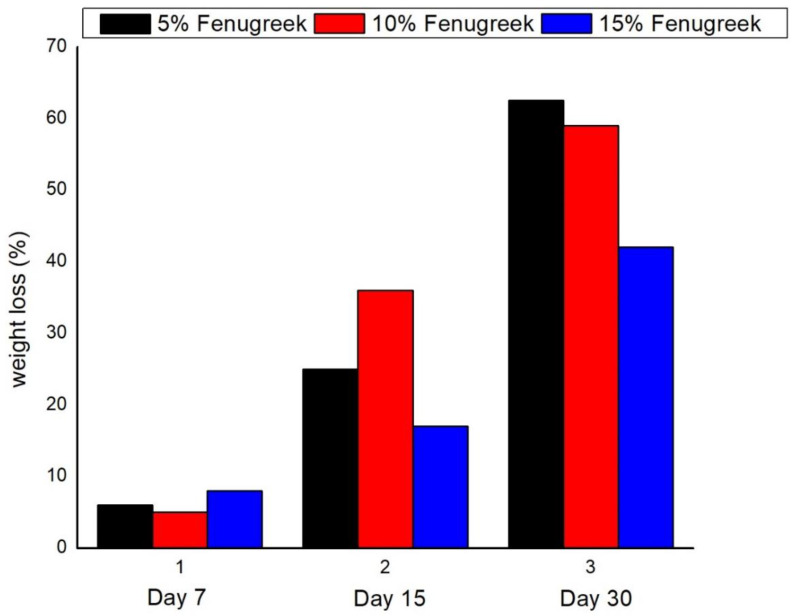
Biodegradibility comparison of the synthesized bio-composites at different percentages of fenugreek.

The biodegradation process of the synthesized bio-composites is represented by Figure 2a–d, Figure 3a–d, Figure 4a–d, Figure 5a–d and Figure 6. The weight loss of the bio-composite films was estimated after 7, 15, and 30 days. Linear biodegradation was observed in the bio-composite samples, which indicated obedience to pseudo-zero-order kinetics in reaction rates and constant, as well as independent rates of biodegradation [35]. From the figures, it can also be seen that the bio-composite films had a slower degradation initially. However, after 30 days the degradation was enhanced. The results also suggest that the addition of fenugreek slowed the degradation process of the starch-based bio-composite films. A sample with a lower percentage of fenugreek was obtained with maximum degradation among all synthesized bio-composite films. Ecologically-friendly and biodegradable properties were proved by the outcomes demonstrated.

### 3.2. Tensile Strength

**Figure 7:**
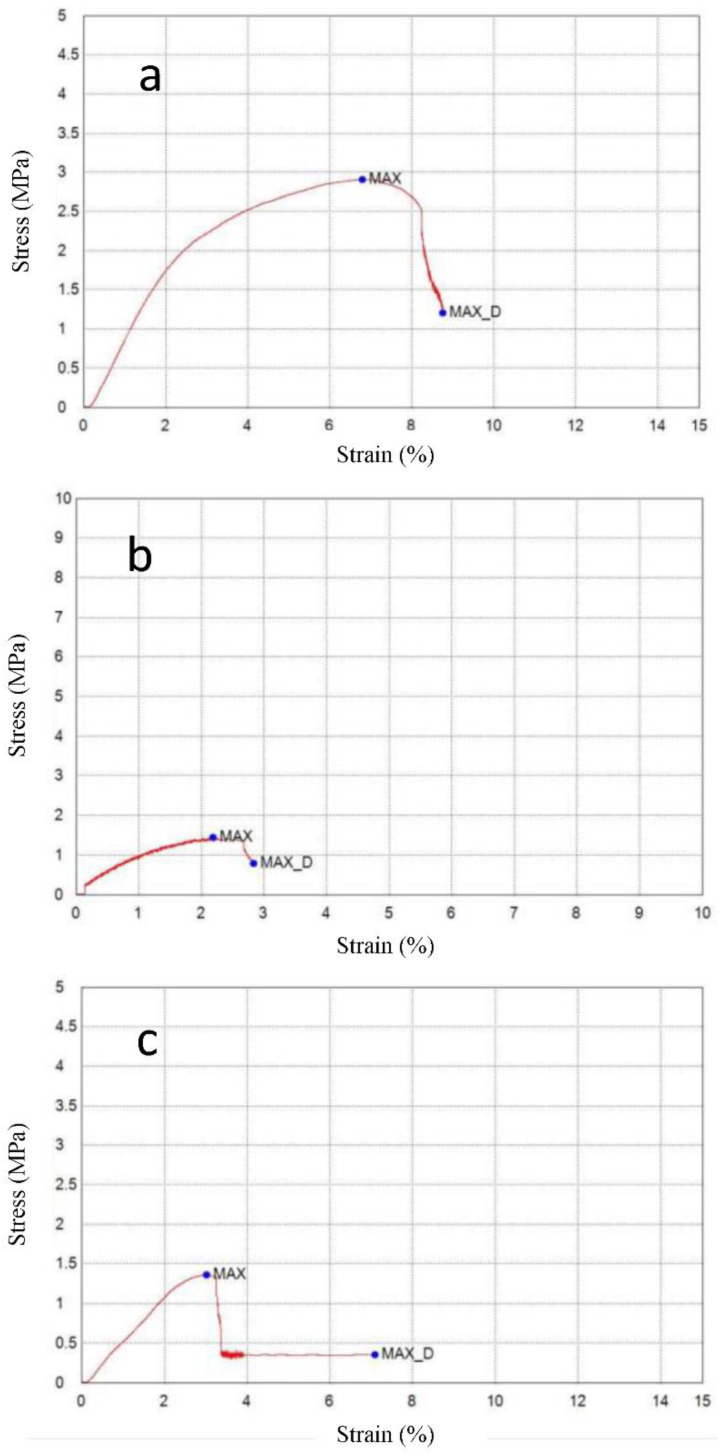
Tensile behavior of the bio-composite samples at various ratios of fenugreek (**a**) 5%, (**b**) 10%, and (**c**) 15%.

Figure 7a–c shows the tensile behavior of the developed bio-composites. Table 4 shows the comparison of tensile strength, tensile strain, and the Young’s modulus of the prepared samples. An increase in the percentage of fenugreek decreased both the tensile strength and the tensile strain of the bio-composites, up to a certain level. Maximum stress development was reflected in the tensile strength of the film during the tensile testing [36]. The higher concentration of fenugreek decreased tensile strength and increased flexibility [37]. The presence of 15% of fenugreek exhibited the lowest tensile strength of 1.357 N/mm^2^, due to the increased water presence in the matrix of the film, provided by the hygroscopic nature of fenugreek. The disarrangement of the polymer network was caused by the fenugreek present in the film, which worked to decrease the tensile strength and increase the film’s flexibility [38]. Lower concentration percentages of fenugreek were distributed properly in the matrix, resulting in higher mechanical strength. However, minimum tensile strain was obtained after the addition of 10% of fenugreek. Tensile strain slightly increased after that percentage.

### 3.3. Scanning Electron Microscopy

**Figure 8:**
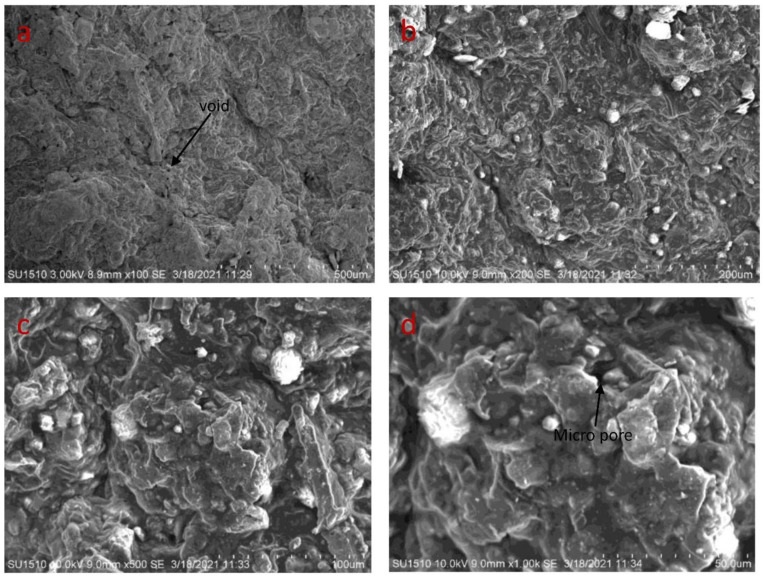
SEM images of 5% fenugreek bio-composite at (**a**) 500 µm (**b**) 200 µm (**c**) 100 µm (**d**) 50 µm.

**Figure 9:**
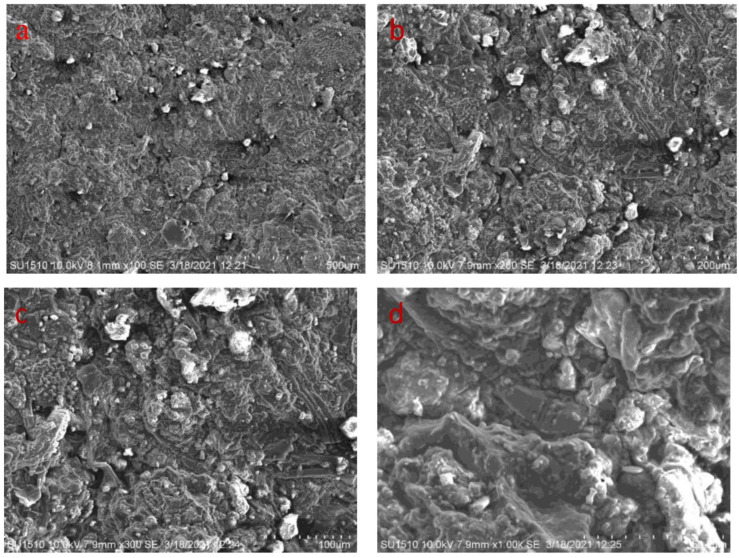
SEM images of 10% fenugreek bio-composite at (**a**) 500 µm (**b**) 200 µm (**c**) 100 µm (**d**) 50 µm.

**Figure 10:**
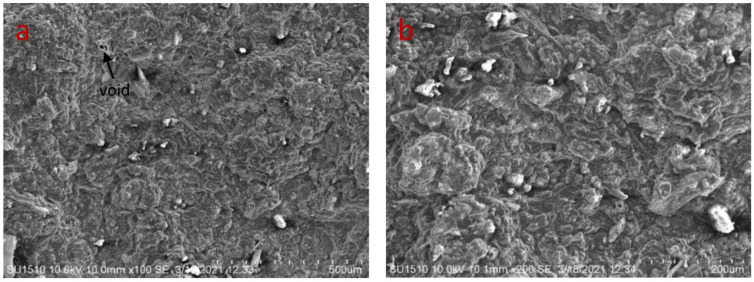

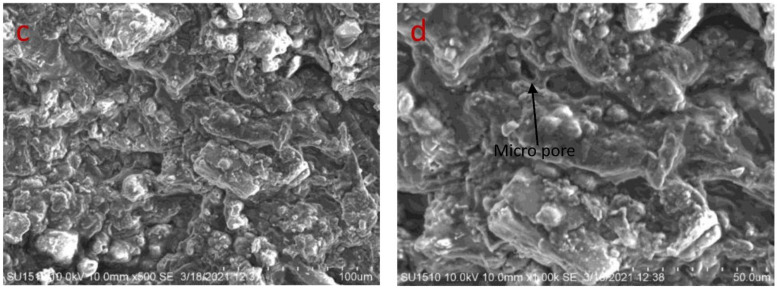
SEM images of 15% fenugreek bio-composite at (**a**) 500 µm (**b**) 200 µm (**c**) 100 µm (**d**) 50 µm.

The microstructure of the developed bio-composites is seen in Figure 8a–d, Figure 9a–d and Figure 10a–d. Defects such as voids, cracks, and micropores are seen in SEM images, and result in more porous and irregular surfaces. The samples containing a higher percentage of fenugreek show more micropores, due to the cohesion between the tamarind starch and fenugreek. Voids and micropores may affect the modulus of elasticity and tensile strength of the bio-composite samples [39,40]. The biodegradation process of the bio-composite samples may be accelerated in the soil, due to the interaction between micropores and microorganisms [41]. Impurities are seen with globular-like fibers on the surface in higher magnification images, because potency is provided to the biomass by the non-cellulosic contained in the natural biomass [42].

### 3.4. X-ray Diffraction Analysis

**Figure 11:**
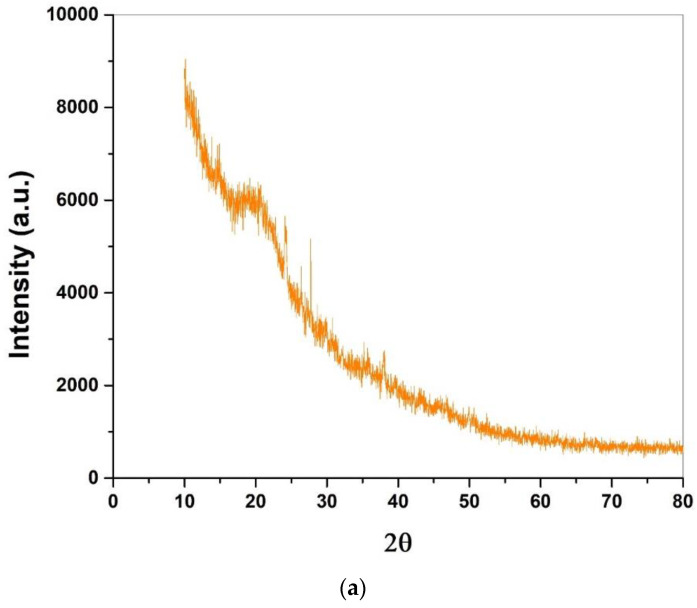

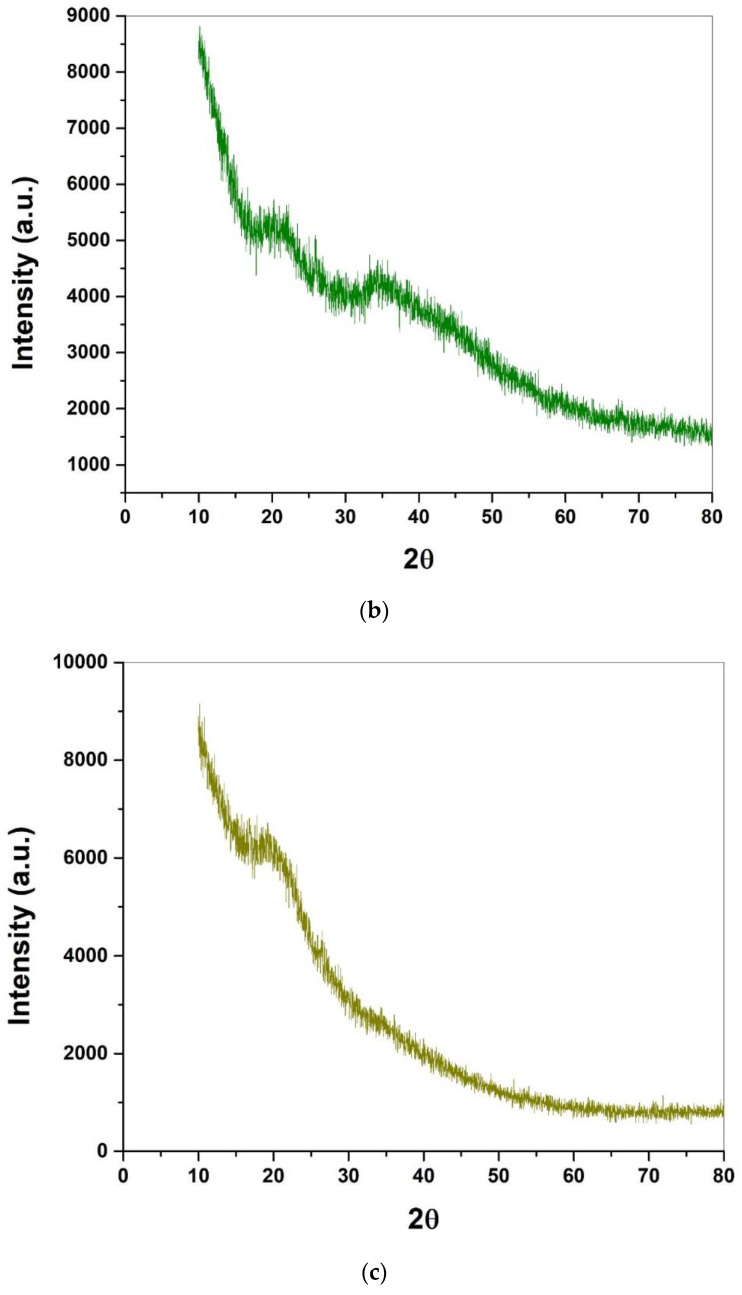
XRD analysis of the bio-composite samples at various ratio of fenugreek (**a**) 5%, (**b**) 10%, and (**c**) 15%.

Figure 11a–c shows the X-ray diffraction patterns of the bio-composite samples with different percentages of fenugreek, where intensity is shown vertically and the 2θ region is shown horizontally. The bio-composite sample with 5% fenugreek shows only two distinct peaks. Small peaks in the region of 2θ are represented in the bio-composite by the XRD pattern. Sharp peaks confirm the crystalline nature of the cellulose [43]. The peak at 2θ = 20° is attributed to the parallel-chain-segment aggregate region, which confirms the bio-composite semi-crystalline nature [44]. The region of 2θ = 28° also relates to the occurred disturbance in the cellulose at the time of the acetylation process, which can cause breakage in the micro-fibrillar structure of the cellulose [45,46]. However, the bio-composite samples with 10% and 15% fenugreek show no peaks, confirming the amorphous structure of the synthesized bio-composite samples.

### 3.5. Thermal Analysis

**Figure 12:**
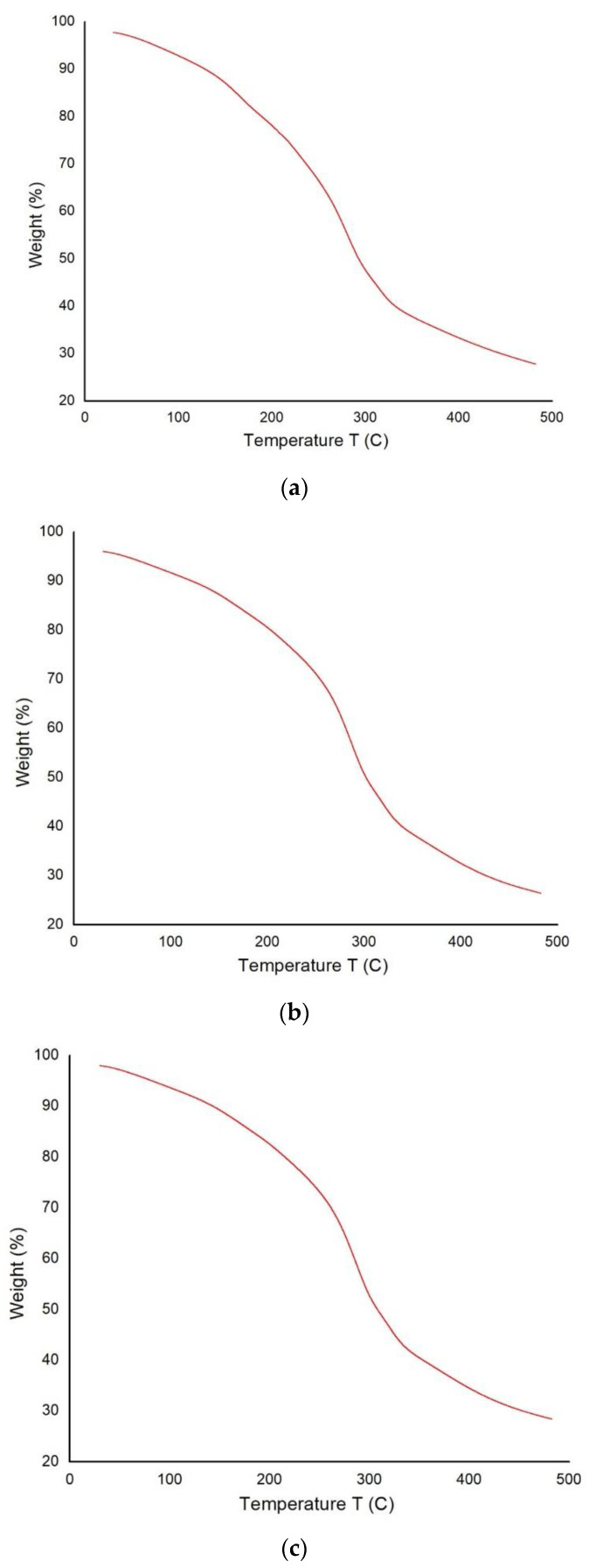
TGA analysis of the bio-composite samples at various ratios of fenugreek (**a**) 5% (**b**) 10% and (**c**) 15%.

Figure 12a–c shows the TGA analysis of the bio-composite samples. Similar trends are observed in all the samples with variable temperatures. However, the graphs indicate that weight loss is reduced, due to the higher fenugreek percentage at elevated temperatures. Initially, 5% of weight loss is observed, because of moisture evaporation as the temperature changes from 25 to 100 °C [47,48]. Moreover, cellulose decomposes rapidly at around 350 °C because of the removal of hemicelluloses. Only about 30% weight is left for the pyrolysis of the cellulose skeleton at 350 °C [49].

### 3.6. Antibacterial Activity

**Figure 13:**
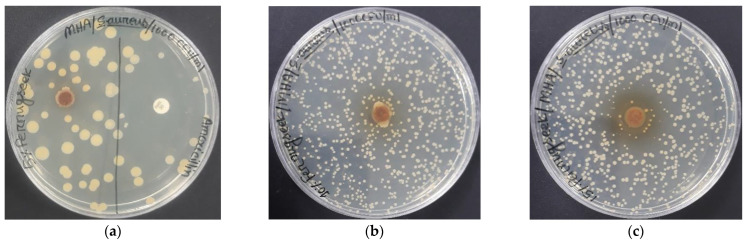
Antimicrobial analysis of the bio-composite samples at various ratio of fenugreek (**a**) 5%, (**b**) 10%, and (**c**) 15%.

The antibacterial analysis of the bio-composite samples against *Staphylococcus aureus* is seen in Figure 13a–c. The samples showed some form of antibacterial activity against *Staphylococcus aureus,* due to the presence of active phytocompounds shown in FTIR analysis. The antibacterial performance was obtained due to the presence of fenugreek. Reducing and/or capping agents containing phytochemicals may be present in the samples, which kill the virus. Moreover, inherent antibacterial activities may also be present in those botanic compounds. In addition, different types of metabolites such as alkaloids, terpenoids, tannins, flavonoids, and glycosides are available in fenugreek. These metabolites have antibacterial properties which make fenugreek active against bacterial strains [50].

## 4. Conclusions

Starch-based bio-composite films with varying percentages of fenugreek were successfully developed, using vinegar and glycerin. The properties of the bio-composite films were significantly affected by the concentration percentages of fenugreek. It was observed from the biodegradability test that a maximum of 62.5% of the bio-composite film was biodegraded in only 30 days. Lower percentages of fenugreek distributed properly in the film resulted in higher mechanical strength. Higher percentages of fenugreek led to the formation of an amorphous structure of bio-composite films, confirmed with XRD analysis. Thermal stability was assured for the synthesized bio-composites by thermal analysis. Some samples showed antimicrobial activity against *Staphylococcus aureus* bacteria, which confirmed the usability of the synthesized bio-composites in packaging applications.

## Figures and Tables

**Table 1:** Percentages of chemical compounds present in fenugreek seed.

Constituent	Moisture	Ash	Fat	Protein	Fiber	NFE
Percentages	10.17 ± 0.06	2.63 ± 0.15	7.47 ± 0.31	27.55 ± 1.56	3.79 ± 0.31	48.39 ± 1.33

**Table 2:** Percentages of chemical compounds present in tamarind seed.

Constituent	Moisture	Protein	Fat/Oil	Crude Fiber	Carbohydrates	Total Ash	Total Sugar
Percentages	9.4–11.3	13.3–26.9	4.5–16.2	7.4–8.8	50.0–57.0	1.6–4.2	11.3–25.3

**Table 3:** The used constituents with their percentages.

Sample	Distilled Water	White Vinegar	Glycerol	Tamarind Seed Starch	Fenugreek Powder
S1	69% (360 mL)	8% (40 mL)	6% (30 mL)	12% (60 gm)	5% (26 gm)
S2	66% (360 mL)	8% (40 mL)	6% (30 mL)	11% (60 gm)	10% (54 gm)
S3	62% (360 mL)	7% (40 mL)	5% (30 mL)	11% (60 gm)	15% (85 gm)

**Table 4:** Comparison of tensile strength, tensile strain and Young’s modulus of the prepared samples.

Sample	Tensile Strength (MPa)	Standard Deviation	Tensile Strain (%)	Standard Deviation	Young’s Modulus (MPa)
S1	2.905	0.110	6.797	0.353	0.427
S2	1.435	0.071	2.189	0.094	0.655
S3	1.357	0.062	3.029	0.125	0.448

## Data Availability

Data will be available upon reasonable request.
